# Towards an understanding of resilience: responding to health systems shocks

**DOI:** 10.1093/heapol/czx183

**Published:** 2018-01-09

**Authors:** Johanna Hanefeld, Susannah Mayhew, Helena Legido-Quigley, Frederick Martineau, Marina Karanikolos, Karl Blanchet, Marco Liverani, Esther Yei Mokuwa, Gillian McKay, Dina Balabanova

**Affiliations:** 1 Department of Global Health and Development, London School of Hygiene and Tropical Medicine, 15-17 Tavistock Place, London, UK; 2 Saw Swee Hock School of Public Health, National University of Singapore, 12 Science Drive 2, #10-01, Tahir Foundation Building, Singapore; 3 European Observatory on Health Systems and Policies, London, UK; 4 Health in Humanitarian Crisis Centre, London School of Hygiene and Tropical Medicine, London, UK; 5 Njala University, PMB, Freetown, Sierra Leone

**Keywords:** Resilience, health systems, Ebola, financial crisis, climate change, migration

## Abstract

The recent outbreak of Ebola Virus Disease (EVD) in West Africa has drawn attention to the role and responsiveness of health systems in the face of shock. It brought into sharp focus the idea that health systems need not only to be stronger but also more ‘resilient’. In this article, we argue that responding to shocks is an important aspect of resilience, examining the health system behaviour in the face of four types of contemporary shocks: the financial crisis in Europe from 2008 onwards; climate change disasters; the EVD outbreak in West Africa 2013–16; and the recent refugee and migration crisis in Europe. Based on this analysis, we identify ‘3 plus 2’ critical dimensions of particular relevance to health systems’ ability to adapt and respond to shocks; actions in all of these will determine the extent to which a response is successful. These are three core dimensions corresponding to three health systems functions: ‘health information systems’ (having the information and the knowledge to make a decision on what needs to be done); ‘funding/financing mechanisms’ (investing or mobilising resources to fund a response); and ‘health workforce’ (who should plan and implement it and how). These intersect with two cross-cutting aspects: ‘governance’, as a fundamental function affecting all other system dimensions; and predominant ‘values’ shaping the response, and how it is experienced at individual and community levels. Moreover, across the crises examined here, integration within the health system contributed to resilience, as does connecting with local communities, evidenced by successful community responses to Ebola and social movements responding to the financial crisis. In all crises, inequalities grew, yet our evidence also highlights that the impact of shocks is amenable to government action. All these factors are shaped by context. We argue that the ‘3 plus 2’ dimensions can inform pragmatic policies seeking to increase health systems resilience.

**Key Messages**

- The ability of health systems to respond to external shocks of different kinds may be one of the key features of resilience.
- Learning from past shocks provides important lessons on how to make health systems more resilient. However, context is important.
- Based on this learning, we suggest an approach focused on action in ‘3 plus 2’ core health systems dimensions, to build resilience.
- To strengthen resilience, interventions are needed in the core areas ‘health information systems’, the ‘funding and financing mechanisms’ and most importantly the ‘health workforce’.
- However, promoting good ‘governance’ and recognizing wider systems ‘values’ are essential to ensuring whether interventions in each block are succeeding.
- Well-integrated and locally grounded health systems may be more resilient to shocks.

## Introduction

The 2014–15 outbreak of Ebola Virus Disease (EVD) in West Africa brought attention to the role and responsiveness of health systems in the face of shock (Moon *et al.* 2015). The initial failure to contain the West African EVD outbreak was attributed in part to the weakness of health systems in countries affected and an inadequate investment in mounting a response (Kieny *et al.* 2014; Moon *et al.* 2015). At the same time, failure was not uniform: some communities and parts of the health system, including individual facilities and services, demonstrated considerable capacity to respond to and withstand the impact of EVD better than others, despite suffering a similar level of funding shortages. This brought into sharp focus the idea that health systems need to be not only stronger but also more ‘resilient’ in responding to acute and chronic shocks (Gilson *et al.* 2017). The need to define and operationalize resilience has since come to be seen as critical, particularly as an increasing number of people live in fragile and post conflict settings (Kieny and Dovlo 2015), spurring research and policy interest (Gilson *et al.* 2017; Kieny *et al.* 2017; Kruk *et al.* 2017).

The concept of resilience has its origins in the fields of engineering (Woods and Hollnagel 2006), environmental science and ecology (Elmqvist *et al.* 2003; Walker *et al.* 2004), where it has developed to suggest that the social systems respond to shocks in a variety of ways: absorbing these, or as a consequence of shocks either returning to their original equilibrium or reaching a new equilibrium (transformative shocks) that makes them more resilient (Holling 1986). In clinical psychology and mental health research the concept is manifested as the ability of the individual to adapt to adverse conditions, trauma or stress (Connor and Davidson 2003; Tugade and Fredrickson 2004). Compared with these developments, the emergence and use of this concept in the health policy and systems and public health literature is relatively recent (Kruk *et al.* 2015) and the implications of the concept for policy implementation remain unclear. There is no widely accepted definition, and resilience is often equated with health systems strengthening. Health systems strengthening is typically viewed as efforts to improve, or strengthen, the system to operate more effectively, efficiently and equitably (De Savigny and Adam 2009). Our working definition of ‘health systems resilience’ draws on the ideas of Blanchet and others that health systems resilience is about the system being able to adapt its functioning to absorb a shock and transform if necessary, to recover from disasters (Blanchet 2015). In this definition, the ability of a health system to respond to external shocks—including but not limited to infectious disease outbreaks and natural disasters such as a tsunami—is seen as one of the key elements of health systems resilience (Kruk *et al.* 2015; HSG 2016).

This article seeks to add to the understanding of health systems resilience, by examining empirical evidence of how health systems responded to past shocks. We use the term shocks to mean stresses and extreme challenges to the system caused by external events. These can be immediate and time-bound, such as a tsunami or a flood affecting a health system, or can unfold over a period of time—such as a financial crisis. Based on this comparative analysis, we identify a set of essential aspects—termed here ‘dimensions’ of the health system—that emerged as key to its resilience in the face of these events.

## Material and methods

In this article, we sought to develop a framework for health systems resilience based on the analysis of four types of contemporary shocks to health systems: the financial crisis in Europe in 2008 onwards; climate change disasters in low and middle income countries; the EVD outbreak in West Africa 2013–16; and the recent refugee and migration crisis in Europe from 2013 onwards (see box 1). The specific ‘shocks’ were selected through purposive sampling, by a group of health systems researchers associated with the London School of Hygiene and Tropical Medicine, who had independently conducted research in each of these areas over the past 5 years. The selection criterion for the set of cases was that resilience of the health system to withstand a crisis was independently identified as a key theme in their analysis. We then sought to identify research cases that represented diverse types of shock, geographic and income settings. The researchers then developed a method (set out below) to comparatively analyse lessons for health systems resilience across these cases. Based on this analysis, we identify lessons about the range of responses that should be considered in strategic planning to enhance health system resilience.

**Box 1. Process of analysis**

We began by defining the core questions to guide our comparative analysis to help enable the development of a framework on health systems resilience. These were: what were the health systems responses to these different shocks in each setting? What factors determined these responses and their success? Were there any common features across these? Which responses made health systems more resilient? and what lessons can be learnt for other countries seeking to equip their health systems to deal with shocks?

Our conceptualization of health systems draws loosely on a range of mainstream frameworks, such as the World Health Organization (WHO) ‘building blocks’ approach (WHO 2007), the framework focused on ‘control knobs’ favoured by the World Bank and developed by Roberts *et al.* (2003), and one incorporating macro-influences and interactions with the health system (Smith and Hanson 2012). We recognize that health systems are not just a sum of blocks but also complex and adaptive systems which are shaped by the decision makers as well as the people working and interacting within them, namely patients and health workers and the communities within which they are located (Gilson *et al.* 2011; Sheikh *et al.* 2014). In this conceptualization, people, processes, systems, power relations and values are an integral and mutually dependent parts of the health systems.

Following an initial review of data on shocks in general, authors undertook comparative analysis by examining the impact of each shock on each of the WHO health systems’ building blocks: health workforce, health financing, health management information systems, products and medicines, health services and governance (WHO 2007). At the next step, the converse type of analysis was conducted; assessing the extent to which each building block had contributed to health system resilience. An extended version of Table 6 was developed as a tool for data extraction, with two or more researchers extracting and synthesising data for each block. Through this analytical process, aspects of the health system that appeared to have been of lesser relevance to resilience in the shocks examined were found to be less important. The analysis focused on aspects identified as core, on identifying interrelationships between blocks and policy process issues. For the purpose of this discussion, we termed the aspects or blocks emerging as critical to resilience ‘health systems dimensions’ to avoid conflation with other health systems models. The outcome of interest in each case was whether the health system had been able to adapt in response of the particular type of shock, and what were the factors that impeded or facilitated this response.

Once the initial findings were identified, the authors systematically reviewed each of these to understand the extent to which these were context-specific, and held wider lessons for health systems in other contexts. The initial results were presented to an expert audience at the Vancouver Health Systems Symposium (14-18 November 2016) for further triangulation.

The term ‘shock’ rather than crisis was chosen as the types of events examined were comparatively short- to medium-term in nature. We recognize that there are many longer-term crises, for example epidemics of chronic disease or underfunding over a period of years which affect and equally require health systems resilience, however these require further analysis (Gilson *et al.* 2017). Similarly, climate change is an ongoing process which is likely to affect disease patterns and food security (hence nutritional illnesses) over a long period of time (Mayhew and Hanefeld 2014)—we focus on specific climate change-related disasters as one-off shock events, e.g. flooding, tsunamis.

Findings presented below are based on this analysis and focus on the impact of the shocks on five key dimensions of the health system and the insights that can be generated for the health system response as a whole.

To ensure maximum learning from our comparative analysis of shocks, we present the results by health system dimension. In addition, we also showcase specific learning and analysis for each of the 3 plus 2 dimensions identified as important for resilience. We present these in shock-specific tables (Tables 1–5), as well as a comparison across shocks (Table 6). For the recent migration crisis in Europe we provide two tables: Table 4 focused on humanitarian crisis and post-conflict aspects, and Table 5 solely focuses on migration and mobility, as these appeared as distinct aspects in our analysis. By presenting the comparative results of our analysis as well as in-depth case studies, we seek to contribute not only to the knowledge base on resilience overall, but also to provide evidence on how each specific type of shock affects health system resilience.

**Table 1.** Response to Ebola according to health system functions and cross-cutting dimensions

	Health systems dimension		
	Health and management information systems	Funding/financing mechanisms	Health workforce
Ebola	Staff in most affected areas and facilities had little time to prioritize data entry and analysis and to use this for decision-making. Military responders set up parallel surveillance systems, raising issues of sustainability. The rapidly evolving response required intense daily monitoring with a few easily measurable indicators. However, these indicators only gave a partial view of the situation (e.g. early national indicators focused on bed numbers, not ambulances or contact tracing line lists). There was little operational consideration of the value that qualitative data had to contribute to making sense of the HMIS.	Large amounts of money from Western governments were dedicated to eradicating Ebola; governments and organizations sometimes found it difficult to absorb this level of funding. The ‘no regrets’ model of donor funding increased the potential for innovative and bold programming, but financial accountability practices meant that the majority of funds went to international rather than national or local organizations. Funds allocated to go through national governments to pay health workers or buy supplies were hamstrung by inadequate financial management systems. In the post-Ebola/recovery stage there continues to be increased donor attention, but with a relatively narrow focus on recovery priorities. There has been a shift away from sectoral areas (e.g. gender empowerment and sexual violence, core health education/training) that are not included in the recovery frameworks.	Facilities affected early in the epidemic sustained far higher staff mortality than those affected later on once training and supplies were mobilized and coordinated. Wide-scale training of national clinical, hygiene and burial staff by international organizations. The ability of the health system to absorb these trained lay-people was limited. In the recovery period international expertise continues to build national capacity in clinical and laboratory research around infectious disease. Comprehensive training of district-based surveillance officers, who in Sierra Leone have taken on other work post-Ebola.
Values (cross-cutting)	Global humanitarian crises and the militarisation of aid, as seen in the Ebola crisis, raises questions around the moral obligation to intervene and the style of such an intervention, in particular the tensions between the humanitarian imperative (altruism) and global health security (self-protection). Additionally, unanswered questions exist about the moral obligations of alerting others to a health threat that may spread beyond household/village/national borders (obligation to others) versus the potential personal sanctions that may result (obligation to self). This public health response required citizens to transgress deeply ingrained moral codes that are critical in day-to-day health, economic and social survival. The need for international expertise to support this work required specialised services for sick international staff to which national staff did not have guaranteed access.		
Governance (cross-cutting)	Early response oversight mechanisms struggled to effectively reconcile epidemic control priorities with wider political and economic priorities. The establishment of top down, military-style command and control operational institutions, with the involvement of national and international military in all three most-affected countries (although in different ways), proved better able to manage these tensions. National and international oversight of the wider impacts of the epidemic and the response was, however, lacking. Local (village/district) governance was often disconnected; local leaders who are important in governance and planning of local responses were not brought in at the outset, although may have been involved later on in varying degrees depending on the country. International and national governance of the ownership of clinical data and biological samples was weak, leading to controversial ‘extractive’ research practices.		
Case-specific lessons learned	Negotiating competing crisis-specific and wider health, social, economic and political priorities remained challenging throughout the response. Critical gaps between local, national and international organizations, particularly in terms of institutional and workforce capacities, seriously undermined the ability to effectively scale up the immediate response and translate this into sustainable capacity building. A failure to situate response interventions in dynamic local social contexts compounded the ineffectiveness of early response efforts. While this improved somewhat as the response evolved, an incomplete integration of social considerations into operational-level decision-making mechanisms led to missed opportunities to improve the effectiveness and acceptability of the response.

**Table 2.** Response to financial crisis according to health system functions and cross-cutting dimensions

	Health systems dimension		
	Health and management information systems	Funding/financing mechanisms	Health workforce
	HMIS are largely unfit to monitor health impacts of economic crises as: (1) there is a delay before population health and health systems performance data become publicly available; (2) changes in population health are not always directly attributable to one crisis due to multiple underlying changes in health determinants; (3) delayed health effects are largely unreported due to difficulties in attribution and interpretation; and (4) no forecasting of financial crisis is considered in planning for health.	Many countries in the EU demonstrated pro-cyclical patterns of public spending on health during the crisis, which made them vulnerable to economic shock. Countries adopted a mix of measures to mitigate budget shortfalls, ranging from explicit cuts to attempts to mobilize public revenue. Yet, public spending on health fell in many EU countries during the crisis. The scale of cuts combined with high levels of OOPs led to worsening of access to care e.g. in Greece and Latvia. Adequate levels of public funding before the crisis helped some countries respond. Automatic stabilizers (built-in countercyclical mechanisms in the form of reserves and formulas for government budget transfers) made a difference in maintaining public revenue for the health system for at least some time. Health coverage was affected, with an increase in user charges and a reduction in entitlements (e.g. access to medicines). However, a few countries increased levels of financial protection for some of the most vulnerable groups.	The crisis had a negative impact on workers’ pay and numbers in many countries in Europe, with substantially reduced wages and staff lay-offs in the hardest-hit countries.
Values (cross-cutting)	The need to respond to economic shocks should be an integral part of health system policy goals. Evidence suggests that the important economic, social and health system benefits of promoting financial protection and access to health services at a time of economic crisis played little (if any) role in fiscal policy decisions. Disadvantaged groups are likely to be first or worst affected in terms of access and OOPs. When data on increases in mortality due to crisis were available, e.g. suicide rates in men, these did not lead to action.		
Governance (cross-cutting)	There is a need for health to connect more closely with wider economic governance, to argue in non-crisis times for greater investment in health and policies aimed at stabilizing health systems and protecting population health during crises. It is important to note that health will not be the leader of these discussions, but rather play a role in integration and advocating for these policies. Greater integration into economic governance mechanisms also enables better forecasting of impending crises.		
Case-specific lessons learned	The most vulnerable populations were worst affected. Responses varied greatly between countries, depending on underlying importance and values surrounding health. Policy responses before and during a crisis had a real impact on systems’ ability to withstand shock.

**Table 3.** Response to climate change according to health system functions and cross-cutting dimensions

	Health systems dimension		
	Health and management information systems	Funding/financing mechanisms	Health workforce
Climate change	Early-warning systems for climate-related natural disasters are in place in some middle-income countries that are significantly and historically affected (e.g. the Philippines). Extreme weather events are increasingly unpredictable and existing models have been unable to predict some disasters e.g. the Haiti tsunami in 2010. Some disease surveillance and population data trends exist but there is no systematic forecasting of more chronic climate-related health changes, or cross-sectoral sharing of information (e.g. on weather events, changing crop and zoonosis patterns) except through *One Health* initiatives.	Climate Investment funds are available through various mechanisms. International Bank for Reconstruction and Development (World Bank) is chairing several of these.	Some initiatives exist under the One Health agenda to better connect animal and human health in terms of workforce preparedness. Attention to effects on health workforce mainly in relation to responding to extreme weather events as a humanitarian or disaster-related emergency.
Values (cross-cutting)	Rich countries are often to ‘blame’ (emissions) while poorer countries are considered ‘victims’, but these lines are blurring with the advent of the emerging economies (e.g. India and China). This ‘blame’ brings with it moral obligations (with varying commitments) by rich countries to support poorer countries in adapting to the effects of climate change, but this agenda is driven by economic development actors not health actors.		
Governance (cross-cutting)	This is the weakest part of thinking to date. There is no inter-sectoral governance, only ad hoc inter-sectoral planning initiatives mostly around One Health and disaster response. The health sector needs to connect to climate change governance at national and global levels.		
Case-specific lessons learned	Development of workable, useful models of inter-sectoral coordination is needed. Experiences from disaster-response approaches to extreme weather events—for example the UNOCHA Cluster system—demonstrate how difficult it is to coordinate and sustain these initiatives. Lessons need to be translated into a continuous systems response in which the health sector is able to map and act on the critical multi-sector links it needs to make to share forecasting information and multi-sectoral response.

**Table 4.** Response to humanitarian crisis/armed conflict and migration and mobility according to health system functions and cross-cutting dimensions

	Health systems dimension		
	Health and management information systems	Funding/financing mechanisms	Health workforce
Humanitarian crisis/armed conflict & migration and mobility	The HMIS in such a shock is often unable to function properly or capture the type of data necessary during armed conflicts (e.g. injury- and surgery-related data). Armed conflicts are challenging as it may be difficult to predict when they will occur, and with what intensity. Some organizations have information and intelligence systems in place that can: (1) monitor rumours to identify tensions between communities within countries (2) provide regional geopolitical analysis to identify potential tensions between countries and any movement of troops (3) estimate the likelihood of armed conflicts, especially in countries that experienced conflict during the last 15 years	Emergency response requires a large amount of funds mobilised in a very short time. Affected countries do not usually have sufficient reserves, so funds need to come from international agencies. Pooled funding is available through various multilateral (e.g. UN agencies, European Civil Protection and Humanitarian Aid Operations) and bilateral (e.g. Disasters Emergency Committee and START Network (UK), Office of US Foreign Disaster Assistance, Qatar Government) mechanisms.	Armed conflicts result in the disruption of health services and the closure of facilities due to populations and health care workers fleeing the region, and violence against healthcare workers by combatants. Emergency responses during armed conflicts are often accompanied by deployment of external health staff and managers to support existing national staff in the delivery of health services.
Values (cross-cutting)	Humanitarian interventions during conflicts are guided by humanitarian principles based on neutrality, impartiality and independence to ensure that all humankind shall be treated humanely and equally in all circumstances, by saving lives and alleviating suffering, while ensuring respect for the individual. This requires offering health services in all regions, and more specifically in regions directly affected by conflicts and those hosting displaced populations trying to find refuge from violence. To facilitate access to health services, free healthcare is offered to affected populations. This has in the past created tensions between displaced and host populations, who do not have access to the same quality of care and have to pay user fees.		
Governance (cross-cutting)	Humanitarian coordination mechanisms are in place through the Cluster approach coordinated by UNOCHA, and to which all humanitarian actors (national and international) are supposed to contribute. In reality, the system is not fully functional, and is often criticised for not being coordinated and for creating inefficiencies in the health system. The emergence of new humanitarian actors from Qatar, Brazil and Korea, and many individual initiatives funded through crowdfunding, make including all actors even more difficult.

**Table 5.** Response to migration and mobility according to health system functions and cross-cutting dimensions

	Health systems dimension		
	Health and management information systems	Funding/financing mechanisms	Health workforce
Migration and mobility	HMIS is not designed to capture mobility or migration status or to respond to these pressures. This is complicated by the fact that migrants find it difficult to hold onto their medical history, so cannot share it with clinicians.	Citizens feel strongly about national health systems, and anti-migrant feelings can be exacerbated by foreigners using health services. Pressure from the general public to stigmatise migrants and exclude them from the mainstream health system is not uncommon.	Clinicians may not speak the same language as newly arrived patients. Migrants may have different expectations of care and find it difficult to accept care from clinicians in the new country. Staff must approach potentially sensitive topics (i.e. family planning and abortion) in a culturally sensitive manner.
Values (cross-cutting)	Migrants may not know their rights and if they are not covered by the humanitarian or new country’s system they may need to pay for health services. In many national health systems entitlement to health services for migrants is not assured.		
Governance (cross-cutting)	The unpredictability of migration can make it difficult for governments to estimate necessary financial and staffing resources. International funding mechanisms are slow and funds are rarely released quickly. There is no current functional mechanism at global level to address mobility/migration between health systems in a way that addresses these concerns.		
Case-specific lessons learned	Likely to increase inequities, as currently no adequate financial mechanisms to address migration and mobility exist at international level. Criminalization or possible financial penalties make monitoring harder as migrants may seek to hide their migratory status. Current responses rely on health workforce initiative and less on systems responses. For systems to become and remain resilient they need to be supported systematically.

**Table 6.** Lessons learnt across shocks

	Health systems dimension		
	Health and management information systems	Funding/financing mechanisms	Health workforce
Lessons across types of shocks	HMIS needs to be linked to broader forecasting trends (e.g. financial crisis and climate change) and is not fit-for-purpose for migration and mobility of populations. Extra capacity is needed to forecast longer-term systems shocks that may not be linked to health. This will require integration with other areas.	**National mechanisms:** Inbuilt stabilisers support resilience to shocks. Government actions do matter. OOP expenditures often increase (possibly due to a lack of knowledge of entitlements or the rescinding of them). Inequalities increase. **International mechanisms:** Where international funding becomes available there is often a duplication of effort, perverse incentive structures and the problems associated with these. There are no good examples of international funding mechanisms that would build greater resilience in national health systems.	Health staff are key to resilience, as they are the first responders. However, they are also the first and often hardest hit by crises. Greater resilience requires completely new and different skills for health workers relating to long-term shocks and a prioritisation of health staff development. Appropriate training for shocks is important for responding to these, but this training needs to include longer-term planning for what happens after the emergency stage is over. Shocks can also present opportunities for transformative change (e.g. political transitions).
Values	Values have an important role in shaping responses and preparedness but are not often considered. Values informing decision-making on health responses are often not driven by health needs or health actors. Values can also be the rationale for deciding to take action or respond in crises.		
Governance	Governance is crucial to resilience across all the explored shocks but there is little thinking on this. This may be because shocks are scary and unpredictable, and preparedness is not easy to fund (e.g. climate change, financial crisis and migration). Humanitarian crises often involve top-down, military-style approaches with the associated problems and parallel structures this creates. New global health emergency architecture must take into account the longer-term governance effects of these responses. Loss of trust in institutions affects governance during times of crisis. To build health systems resilience, health actors must engage at higher levels of governance, and must accept that health may not be the lead in these processes.		

## Results

### ‘3 plus 2’ health system dimensions essential to responding to shocks and fostering resilience

**Figure 1.**
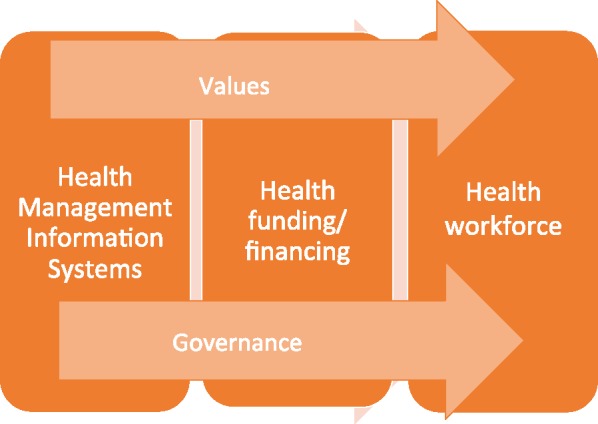
Learning from shocks: a new approach to health systems resilience.

We identify ‘3 plus 2’ critical dimensions of particular relevance to the health systems’ ability to adapt and respond to shocks; actions in all of these will determine the extent to which a response is successful. The three core dimensions corresponding to three health systems functions or building blocks (WHO 2007) are: ‘health management information systems’ (having the information and the knowledge to make a decision on what needs to be done); ‘funding/financing mechanisms’ (investing or mobilising resources to fund it); ‘health workforce’ (who should do it and how). All three dimensions are shaped by two cross-cutting aspects: ‘governance’, as a fundamental function affecting the operation of all system dimensions; and predominant ‘values and beliefs’ shaping the response to the shocks, and how this response is experienced at individual and community levels. We discuss each of these in turn, providing examples of ways in which they contributed to health systems resilience before, during and in the aftermath of the shocks studied. Despite identifying emerging patterns, we recognize that each health systems shock is context-specific, and responses will be determined by a unique mix of health system and external capacities (Smith and Hanson 2011; Mills 2014; Gilson *et al.* 2017). Yet, that learning from past shocks can only help to identify generic factors that help or hinder responsive health systems. The analysis covers mostly short- and medium-term responses, and each shock is likely to have further, possibly far-reaching implications for other aspects of health systems and the population affected (WHO 2007) (Figure 1).

### Health management information systems

The health management information system (HMIS) emerged as crucial to the capacity of health systems to respond to shocks. A comprehensive and well-functioning surveillance infrastructure in particular, including early warning systems, is recognized to be essential to contain disease outbreaks in a timely manner (Kruk 2008). It can also be developed with the intention to enable forecasting and preparing for shocks where these are imminent. However, many of the contemporary HMIS are not fit for purpose, for example their ability to collect and integrate data from mobile and migrant populations is often limited. (Chetty *et al.* 2017).

Importantly, research on climate change and the financial crisis highlights the need for greater integration of HMIS with information systems of other sectors. The change in climate, for example, means that routine health data collection needs to integrate forecasting of extreme weather events and their adverse health consequences, as well as planning for longer-term changes in disease ecology and illness patterns as a result of changes in temperature, precipitation and flooding (Mayhew and Hanefeld 2014). The global financial crisis has demonstrated the extent to which an economic shock can have a worsening effect on population health, as well as on health system performance (Karanikolos *et al.* 2013) with adverse consequences for access to services and affordability. However, the data needed to assess the impact on health and affordability, for example appropriate population health indicators and national health accounts in a timely manner lag years behind, in contrast to the financial and economic sectors data, which are often reported in real time (McKee *et al.* 2012).

Furthermore, the information most needed during an acute crisis may not always be the same as that required for operational purposes and routine management (Bell *et al.* 2012). A health system that is able to effectively and flexibly draw on diverse sources of information, assess the implications of wider societal events outside of the health system and meaningfully integrate the analysis into operational decisions, is therefore crucial to inform adequate short- as well as long-term responses. Since it is unrealistic to expect that the health system of any country will have sufficient capacity and resources to integrate the full spectrum of forecasting and monitoring functions, a more realistic step forward may be to establish information platforms and processes to enable different sectors to share and integrate relevant information, which can inform health system planning and preparedness, including forecasting and procurement of changing drugs and supplies requirements and any accompanying changes in supply and transport (cold) chains.

### Level of funding and financing mechanisms

Our research identified the nature of the funding and financing mechanisms as a core aspect enabling or hindering health systems’ ability to respond to a shock. Based on the analysis of past shocks, we distinguish between national and international/global levels. At the national level, and in relation to different types of shocks, we observe that systems, which are adequately funded are able to better withstand shocks, while gaps in the level and predictability of financing exacerbate the negative impact (Karanikolos *et al.* 2016). Moreover, private expenditure tends to increase, which reflects acute shortages of medications and a lack of awareness of entitlements, thus an increasing reliance on individual and household-centred coping strategies (Karanikolos *et al.* 2013). This is often a result of the introduction of out-of-pocket (OOP) payments as a short-term solution to boost health systems revenues and cover budget shortfalls (Karanikolos *et al.* 2013). During the financial crisis in Europe, many countries opted to introduce OOPs for specific services or increasing the existing ones (e.g. Greece and Portugal), as well as removing subsidies for certain population groups (e.g. Ireland) (Legido-Quigley *et al.* 2013; Kentikelenis *et al.* 2014; Thomson *et al.* 2014). In contrast, community care centres established towards the later stages of the EVD outbreak in Sierra Leone and Liberia were valued because of the free health services they offered for people ‘without’ Ebola, more so than for their care of those diagnosed with EVD (Oosterhoff *et al.* 2015; Oosterhoff *et al. 2015*). In cases, where supply chains are disrupted due to disaster or conflict, cost of medication may increase or medication may only become available through private or informal providers. During humanitarian crises, affected populations tend to have access to free healthcare provided by humanitarian agencies although, during the last 5 years, refugee populations from middle income countries have had to contribute to their health expenditure for secondary care even where medicines were free (Hanefeld *et al.* 2013; Doocy *et al.* 2016). In countries, where the population has formal access to emergency services or primary health care, bureaucratic barriers or fears of deportation may also prevent them from using services. At the same time, the responses to shocks examined, particularly to the financial crisis, also demonstrate that initial adequate levels of funding and in-built counter-cyclical stabilising and reserve accumulation mechanisms for health systems financing can provide a temporary buffer (Karanikolos *et al.* 2016). Finally, specifically for funding and financing mechanisms—maybe more so than for any of the other dimensions discussed here—government action and policies matter (McKee *et al.* 2012) and were shown to have a direct effect on the health systems’ ability to withstand shocks (Thomson *et al.* 2014).

The ability of a health system to rapidly absorb large increases in financial and material resources resulting from efforts to respond to the shocks may also pose a challenge (Green 2016). During crises, this is one of the critical factors that can lead to the local capacity being overstretched. In terms of international or global funding, such as the EVD outbreak, earthquakes or tsunamis or responses to the refugee crisis in Europe, parallel funding mechanisms are often established (e.g. through the Disasters Emergency Committee) to enable fast international mobilisation and deployment of resource. While these parallel systems did allow the rapid recruitment of national and international staff and volunteers, poor existing financial systems and caution to avoid fraud and corruption meant that many national staff in Sierra Leone were not paid for several months while the requisite bureaucratic checks were completed. Not only is this unjust in itself, given the health and social risks faced by the emergency response staff, but the systemic underpayment undermined response efforts by seriously demotivating frontline staff and diverting them away from priorities such as disease control activities. The visible magnitude of the influx of financial and material resources during the EVD epidemic also led to perceived vested economic interests in the continuation of the outbreak. For example, some people interpreted the low but prolonged transmission rates during the ‘long tail’ of the epidemic as evidence that frontline responders were actively complicit in perpetuating the epidemic in order to continue receiving ‘Ebola money’ (Shepler 2016). Thus, the strength of financial and audit systems during crises depends not only on their technical quality but also on their social legitimacy, suggesting that values are fundamental to all health system aspects.

There are few examples of successful international funding mechanisms that enable national health systems to respond to shocks or a crisis, although new mechanisms are currently being developed, such as the new emergency pool fund created by the World Bank to respond to outbreaks (World Bank 2016). The lessons learned indicate the importance of decision makers considering the consequences of different funding mechanisms in their specific context, and weigh in the value of these mechanisms in responding to short-term shocks *vis-à-vis* the longer-term negative effects.

In this respect, it is also important to highlight the overall effects of donor funding and some of the conditionalities attached, such as caps on continuous costs which characterise much of health systems’ investments, on health systems resilience (Stubbs *et al.* 2017). To build health systems resilience this needs to be considered as an important facet of overall development assistance strategies in the longer-term.

### Health workforce

The health workforce is not only essential to a health system’s response to shocks, but in many cases frontline health care workers themselves are amongst the most vulnerable individuals (Fauci 2014; Gostin and Friedman 2015). The health workforce comprises staff at different levels, from frontline clinical workers through to national policy makers, working within a range of different sectors—usually the state, formal and non-formal private and non-profit sectors. We argue that the most effective health workforce response to a shock requires collaboration and coordination across different sectors in such a way as to draw on the particular added value of each. In reality, the distinctions between the above roles and sectors are particularly blurred during an acute crisis where health workers may shift between roles and sectors in response to a rapidly changing context (see box 2). It is nevertheless helpful to consider the characteristic values and limitations of each sub-sector in turn.

The public sector delivers the bulk of health services in many countries. State health workers are often, therefore, the first responders to a crisis. Yet distribution across the country is often inadequate to meet the unexpected needs of an acute crisis (Lehmann *et al.* 2008). An *ad hoc* redistribution of staff to address shortages during acute shock has a knock-on effect on the provision of health services in the country as a whole. Furthermore, many state sectors face institutional and resource constraints to creating new or redeploying existing health worker positions. Nevertheless, with the right management systems in place, the public sector may be the provider best placed to absorb and effectively coordinate increases in health workforce capacity over the medium-term.

The non-profit sector, consisting of a range of organizations from small local charities through to large international non-governmental and multilateral organizations (e.g. UN agencies), is in many ways well-suited to responding to acute shocks. Their institutional ethical mandate, specific technical capacities, the moral profile of their workforce and their risk profile will often align with what is most needed to respond to a major crisis. With appropriate and sufficiently flexible funding, their managerial systems often allow quicker shifts and rapid hiring, deployment and reallocation of staff than equivalent state institutions, although individual organizations may struggle to absorb any substantial increase in staff or finance capacity in the short-term. However, coordination between these organizations and the public sector is crucial to ensure resources are used most efficiently in the short-term and that crisis-specific responses contribute sustainably to health systems strengthening in the longer-term (Chen *et al.* 2004).

Private, for-profit providers include a diverse range of health workers, ranging from employees of large hospitals, through small-scale or non-formal independent biomedical practitioners, to non-biomedical ‘traditional’ practitioners. As such, their response to crises is likely to be highly variable. In resource-poor settings, most private providers work outside the formal health care system, are neither supplied nor monitored by the health authorities, and may not be included in any official register. Such autonomy may be crucial in allowing smaller providers to continue operating in the face of a breakdown in wider financial and logistical systems. On the other hand, a fragmented landscape makes coordination of roles and scaling up essential supplies extremely challenging. Integration into formal response mechanisms may be further hindered by differences in ideological and health beliefs, with state and non-profit providers potentially suspicious of the motives of for-profit institutions, and with biomedical practitioners unwilling to engage with alternative medical practices (Hewlett and Amola 2003).

Efforts to increase health systems resilience should therefore include a central focus on state, non-governmental organizations (NGOs) as well as private health workers. The health workforce at all levels needs the skills and institutional environment to be aware of, and able to respond dynamically and flexibly to, abrupt shifts in the health needs and social context of their patients. Strengthening cross-sector governance mechanisms, coupled with more collaborative relationships between sectors and with society more broadly, is essential to ensuring that sectors work in a complementary way rather than in parallel. Given the often cyclical nature of (some) shocks, this should include longer-term planning to ensure retention and continued support for preparedness efforts once the emergency stage is over.

Box 2. The health workforce at the frontline: a case study from Ebola

At the start of the Ebola response health care workers were among the most vulnerable individuals, with 12% of infections occurring in this group across West Africa in July 2014 (WHO, 2015).

**Public health workforce**

During the first months of the West African Ebola epidemic most health care (Ebola and non-Ebola) was provided by government healthcare workers, even though facilities were undersupplied and understaffed. A number of government facilities closed completely, many staff were unwilling to treat potential Ebola cases and crucially staff were not redistributed to meet the rapidly rising demand in so-called Ebola hotspots. With external support, improved training programmes, supply chains, logistics and financial systems were put in place. When coupled with the temporary employment of large numbers of additional health workers, state facilities were eventually able to mount an effective response against Ebola while continuing to provide routine non-Ebola care

**Non-profit workforce**

While a small number of international NGOs, notably MSF, were able to scale up rapidly in the early stages of the Ebola epidemic, many NGOs that were already operational in-country paused their operations or evacuated their international staff when they were most needed. Despite the ready availability of donor funding from September 2014, many international NGOs were slow to take on health roles during Ebola due to challenges in acquiring suitable employee insurance and concerns that they held insufficient technical expertise in the management of haemorrhagic fevers (whether they were asked to take on direct care or supportive work including the complex water and sanitation needs). Once established, however, non-profit organizations provided a large proportion of Ebola-specific clinical care as well as providing crucial support to personnel across the response efforts.

**For-profit workforce**

During Ebola, most larger for-profit hospitals closed completely, often out of concern for their staff, while many smaller clinics continued to serve patients. Indeed, in Sierra Leone the Ministry of Health and Sanitation mandated that such private providers stop operations as they were unable to guarantee the safety of the care they provided, though the efficacy of such policies was variably adhered to. This group also included the traditional healers, alternative medicine practitioners who were not integrated into the response until very late, despite them often being the first care practitioner that many Sierra Leoneans seek out, especially given the fragility and lack of trust people had in the formal system.

### Governance as cross cutting function

Across all cases examined here, governance of health systems is essential for effective and appropriate responses to shocks. WHO recognizes governance as a cross-cutting health systems function, influencing the operation of all building blocks, yet during crises its role is often overlooked (Gostin *et al.* 2016).

In emergency situations, governance arrangements tend to be top-down, often with multiple parallel international management structures set up or imposed in addition to national government and its structures. Thus, the emergency response to EVD in West Africa was predominantly ‘command and control’ in nature and later co-led by the military (Kamradt-Scott *et al.* 2016). While there may be short-term value in a focused programme of expansion as the only workable solution in the context of an uncontrolled and rapidly expanding epidemic such as with EVD, early and explicit consideration for how to mitigate the harmful wider effect of this approach remains crucial. Importantly, such a hierarchical governance structure is seen as helpful in coordinating the effective distribution of rapidly scaled up human and physical resources. Yet it also limits the extent to which the knowledge, experiences and values of those most affected by the crisis, and those most involved with the response implementation on the ground, can be taken into account at the operational level (Chandler *et al.* 2015).

Good governance also requires horizontal processes of coordination and defragmentation at the national and subnational levels. Although programme interventions to improve health system governance may lead to coordinated management and accountability during a shock, these may be insufficiently aligned with wider governance structures and processes in the broader health system and beyond. For example, during other humanitarian crises, including responses to extreme weather events, coordination mechanisms such as the Cluster mechanism through UNOCHA and other mechanisms have often functioned in parallel to other international responses, and they have either had limited links and relationships with national systems, or were in competition with these (Akl *et al.* 2015; Bourdeaux *et al.* 2015). At the subnational level, it is argued that a higher level of system integration, for example integrating actions between building blocks, and disease-specific services with the broader system, may promote resilience (Mayhew *et al.* 2014). The assumption is that one component may fail but its functions are subsumed into another structure, with critical processes sustained until stability is reached.

Effective governance for response to shocks needs to encompass policy development and action plans beyond the health system. In most examples, we considered, the consequences for health needed to be taken into account at the higher levels of governance and decision making, at national and international level, especially when health shocks transcended national borders, but this was not always the case (Moon *et al.* 2015). The cases of the shocks posed by the financial crisis and climate change illustrate this particularly well, as policies in the range of sectors are seen to have clear implications for social determinants of health, but these are not always brought within a common decision space. Governance of the intersections between the effects from a shock in one sector (e.g. finance) on the outcomes of another (e.g. health) has been neglected despite the clear inter-sectoral implications of the Sustainable Development Goals; this critical aspect of effective governance for building systems resilience is frequently the most underdeveloped dimension.

Good governance and the level of accountability and transparency have implications for the perception of health systems, for example, for whether these are perceived as responsive and trustworthy (Gilson *et al.* 2005).

In sum, evidence examined very strongly suggests that governance is a vital and often neglected dimension when seeking to respond to shocks. Clear lessons here are that both top down and bottom-up approaches are required. These need to be coordinated and integrated as much as possible. Integration with the broader system is likely to promote resilience. The intersectoral governance in particular—going beyond the health sector alone is vital when responding to shocks.

### Values underlying the response

A focus on inputs or structures (such as the WHO blocks) has insufficient recognition of how health policy development, prioritization and health system structures and processes are shaped by the values, beliefs and preferences of the actors within a health system. The role of the underlying values is even more important in crisis situations where they critically shape the ability of health systems to respond to shocks and the nature of that response. ‘Values’ is used here to encompass a range of dimensions, including the political priority given to health during an external shock as well as societal values in which the health system and its workers are embedded, and the personal, professional and societal moral landscapes that play a particularly important role in how difficult decisions are negotiated and compromises reached.

During the financial crisis, some of the health effects (such as increase in suicides) and effects on the health system were anticipated by policy decision makers or became apparent early on Stuckler *et al.* (2011), yet these were not the most important factors motivating the responses to the crisis. Similarly, when natural disasters, such as hurricanes or earthquakes occur, humanitarian aid often focuses on food, clothing, rescue and emergency medicine; health promotion measures may only receive attention after cholera has already broken out.

Values often become more explicit in the response to a health emergency; the reason for intervention is often publicly stated, to attract support and facilitate action. The decision by the United States military to intervene on Ebola was influenced by an underlying belief in the system of military humanitarianism, while responses to the refugee and migrant crisis in Europe have been heavily informed by underlying values and perceptions of military personnel as aggressors (i.e. warring parties) rather than humanitarian agents. At the same time, it is important to recognize the extent to which international humanitarian interventions shape and interact with local values shared by health workers, patients and communities. These externally driven interventions depend on shared understanding of the value of health and may raise the question of whose health is prioritized in the face of shocks. Findings from the financial crisis demonstrate that it was often already marginalised communities whose access to services was curtailed further (Legido-Quigley *et al.* 2013).

Whilst values are important at a societal level, it is also important to recognize that people operating within health systems and those providing and receiving services may hold different values and expectations (Gilson *et al.* 2017). For example, key values in a health system, in addition to the ones related to an effective, safe and good quality system, include equity (justice and fairness), compassion, dignity and respect (WHO 2015). Trust can be important; for example, trust in the health workers in the system in Sierra Leone during the EVD outbreak proved critical in ensuring access to care (Gilson 2005; Kieny and Dovlo 2015). Drawing on the financial crisis, but equally the outbreak of EVD in West Africa and the recent migrant crisis in Europe, it is evident that the level of trust in public (including health) institutions may be key to the ability of health systems to withstand shocks. Trust affects the relationship between the people and the health system, including whether and how people access and use health services, what information citizens are willing to share with the government and whether health workers are responsive to local needs (Hanefeld *et al.* 2017).

Health worker compassion was evident in some European countries when their governments opted to exclude undocumented migrants from the health system: healthcare professionals continued providing services to this group arguing it was against their ethics and core values as healthcare professionals (Legido-Quigley *et al.* 2013).

## Discussion

### Reflections on past shocks: where do we start in building resilience?

In this article, we seek to advance the discussion on how health systems can respond to shocks and, therefore, be more resilient, by presenting a framework to study resilience based on a review of four recent shocks. We focused on blocks and interactions within the health system, which emerge as key dimensions that need attention if health systems are to become more resilient—not merely stronger. This means understanding what dimensions will be key to enabling a system to adapt and if necessary reconfigure, to survive a shock and continue to deliver adequate health care. We take stock of the lessons from a series of past crises: the financial crisis in Europe from 2008 onwards; climate change disasters; the EVD outbreak in West Africa between 2013 and 2016; and the current refugee and migration crisis in Europe. Learning from these past shocks was imperative, specifically in understanding how health systems responded to these crises, whether some of this response was suboptimal, and identifying lessons on what can be done to ensure that responses to shocks are designed and implemented with the objective of building resilience.

We argue that a ‘3 by 2’ approach to understanding health systems’ resilience may facilitate learning from the past and preparing for the future. At the policy and programmatic level, this approach places emphasis on intervening in core areas (‘building blocks’) of the health systems: ‘health information systems’, the ‘funding and financing mechanisms’ and most importantly the ‘health workforce’. However, promoting good ‘governance’ and recognizing and aligning policy with ‘values’ underpinning health systems affect whether interventions in each block are succeeding. Without better understanding of and actions to address the latter two cross-cutting dimensions, strengthening and building the capacity of programmatic responses may be futile. The approach, we offer here helps to identify areas where our knowledge on resilience is more developed and where gaps in knowledge are greatest, specifically on governance and values. However, we note that this framework reflects the learnings from the cases we examined, and it is not intended to be rigid; as evidence increases, new dimensions may need to be added.

Although we identify common patterns across different types of shocks, the importance of context, however, needs to be recognized when planning and implementing responses (Walker *et al.* 2004). The nature and severity of the shocks as well as the pre-existing capacity of the health system to adapt will all influence the nature of the health systems response. Some shocks, for example earthquakes, can—or should—be predicted but this is not true for every country or every event. Ebola appeared in West Africa where it was not known to have occurred before (Moon *et al.* 2015). The financial crisis affected different countries in different ways, and each required a different response. The ecology and society literature distinguishes usefully between the capacity to absorb a shock but continue to function as before; to adapt to a shock—largely functioning as before but with some change; or to have to transform completely in order to survive (Blanchet *et al.* 2017).

It is important also to recognize that the reality may be more complex than responding to one crisis at a time. When a country experiences multiple shocks including political and economic crisis, followed by conflict or disease outbreaks, their effect on the societies, population and health systems is often compounded (Gilson *et al.* 2017). Sierra Leone has experienced war and Ebola; likewise, Zimbabwe has experienced a series of financial and political crises, while also being heavily affected by human immunodeficiency virus (McCoy *et al.* 2008; Martineau *et al.* 2017). To further advance thinking on systems resilience will therefore require consideration of how to adapt and absorb repeated shocks and to understand the pattern by which these waves occur and affect health.

The need to better understand the relationship between health systems’ dimensions (of financing, information systems, human resource planning etc.), and of the health system into wider systems (e.g. ecological system, socio-political system) is also apparent from the analysis of the different shocks. There are indications that integration itself may augment the health systems’ ability to withstand shocks, so that well-integrated health systems that were internally coherent appeared to have greater resilience to shocks (Mayhew *et al.* 2014).

Across the crises studied, we observed that inequalities often increase during and in the aftermath of shocks, whether in terms of health impact, OOPs or access to the system. These need to be mitigated against and considered in all policies seeking to build resilience. An emerging theme across the four cases is that government action has a critical role in implementing and coordinating responses to shocks, and this has an important influence on systems’ resilience and health impacts of a crisis.

Equally, responses to shocks need to be informed by, search for and draw on local resilience in terms of local responses (Richards 2016). The community responses to Ebola and the popular movements in response to the financial crises have demonstrated the power of communities and how they can turn the tide, and systems need better ways to connect and harness them (Wilkinson *et al.* 2017).

We recognize that an effective and appropriate response to shocks is only one aspect of resilience; further developmental work is needed to identify its multiple aspects. The environmental science and management literatures suggest that while recovery from shocks and adaptation is a step forward, complex systems may reach a new equilibrium, characterised by new types of challenges and crises (Carpenter *et al.* 2001). Where ‘adaptation’ refers to the ability of an existing system to change (one or several aspects, and possibly temporarily) to withstand shocks, a new equilibrium literally refers to more fundamental change resulting in a novel, permanently changed characteristic of the system. For example, it is important to recognize that shocks can also present opportunities to regenerate social systems and reach new equilibrium (for example, reform programmes initiated during political transitions) (Carpenter *et al.* 2001; Dalziell 2004). This is demonstrated in other work emphasising the key role of catalysts—which can be disruptive factors, political crises, environmental disasters—as triggers for health systems’ development through opening up political windows of opportunity to intervene and mobilising resources and ideas (Balabanova *et al.* 2013).

In sum, learning from shocks during the recent past provides important insights and opportunities for learning on how to make health systems more resilient. We have set out our framework to facilitate efforts towards greater health systems’ resilience. Effective responses to shocks is only one element of resilience, and more research is needed to understand how health systems move from effective responses to shocks, to broader system reconfiguration and improved resilience (Carpenter *et al.* 2001).

*Conflict of interest statement*. None declared.

## Funding

Funding received from MRC/HSRI grant number MR/N015754/1, Wellcome Trust grant number 102919/Z/13/Z and ESRC grant no. ES/K009990/1.
