# Towards an understanding of resilience: responding to health systems shocks

**DOI:** 10.1093/heapol/czy087

**Published:** 2018-12-04

**Authors:** Johanna Hanefeld, Susannah Mayhew, Helena Legido-Quigley, Frederick Martineau, Marina Karanikolos, Karl Blanchet, Marco Liverani, Esther Yei Mokuwa, Gillian McKay, Dina Balabanova

*Health Policy and Planning*, doi: 10.1093/heapol/czx183.

The authors wish to inform readers that one source of funding (Wellcome Trust grant number 102919/Z/13/Z) was originally omitted from this article’s funding statement. This has now been corrected online.

